# A Simple Technique for Three-Dimensional Imaging and Segmentation of Brain Vasculature Using Fast Free-of-Acrylamide Clearing Tissue in Murine 

**DOI:** 10.22074/cellj.2019.5684

**Published:** 2018-11-18

**Authors:** Arezoo Khoradmehr, Fahime Mazaheri, Morteza Anvari, Amin Tamadon

**Affiliations:** 1 Research and Clinical Center for Infertility, Yazd Reproduction Sciences Institute, Shahid Sadoughi University of Medical Sciences, Yazd, Iran; 2 Department of Biology and Anatomical Sciences, Shahid Sadoughi University of Medical Sciences, Yazd, Iran; 3 The Persian Gulf Marine Biotechnology Research Center, The Persian Gulf Biomedical Sciences Research Institute, Bushehr University of Medical Sciences, Bushehr, Iran

**Keywords:** FACT, Rodent, Three-Dimensional Imaging, Tissue, Vasculature

## Abstract

**Objective:**

Fast Free-of-Acrylamide Clearing Tissue (FACT) is a recently developed protocol for the whole tissue three-dimensional (3D) imaging. The FACT protocol clears lipids using sodium dodecyl sulfate (SDS) to increase the penetration of light and reflection of fluorescent signals from the depth of cleared tissue. The aim of the present study was using FACT protocol in combination with imaging of auto-fluorescency of red blood cells in vessels to image the vasculature of a translucent mouse tissues.

**Materials and Methods:**

In this experimental study, brain and other tissues of adult female mice or rats were dissected out without the perfusion. Mice brains were sliced for vasculature imaging before the clearing. Brain slices and other whole tissues of rodent were cleared by the FACT protocol and their clearing times were measured. After 1 mm of the brain slice clearing, the blood vessels containing auto-fluorescent red blood cells were imaged by a z-stack motorized epifluorescent microscope. The 3D structures of the brain vessels were reconstructed by Imaris software.

**Results:**

Auto-fluorescent blood vessels were 3D imaged by the FACT in mouse brain cortex. Clearing tissues of mice and rats were carried out by the FACT on the brain slices, spinal cord, heart, lung, adrenal gland, pancreas, liver, esophagus, duodenum, jejunum, ileum, skeletal muscle, bladder, ovary, and uterus.

**Conclusion:**

The FACT protocol can be used for the murine whole tissue clearing. We highlighted that the 3D imaging of cortex vasculature can be done without antibody staining of non-perfused brain tissue, rather by a simple auto- fluorescence.

## Introduction

Three-dimensional (3D) imaging has enabled the study of systems from various cellular and extracellular structures, such as vasculature structure or neuronal networks in the brain (1, 2). Such studies require an extremely transparent tissue for the detection. Different protocols have been developed for the whole tissue clearing and 3D imaging. Benzyl alcohol and benzyl benzoate (BABB) were the first to make fixed tissues as thick as 2 cm transparent for the deep microscopic imaging compared to <50 µm using conventional immunohistochemical techniques (3). Several advances have been made for a high-resolution and a large-scale imaging of cleared tissue, including Scale (4), dibenzyl ether (DBE) (5), three-dimensional imaging of solvent-cleared organs (3DISCO) (6), See Deep Brain (seeDB) (7), ClearT (8), Clear Unobstructed Brain/Body Imaging Cocktails (CUBIC) (9), System-Wide control of Interaction Time and kinetics of Chemical (SWITCH) (10), and ultimate DISCO (uDISCO) (11).

Considering the limitations of the mentioned techniques including, fluorescence quenching of samples, incomplete clearing specimens, and lack of feasibility for antibody labeling, a series of other techniques have been developed. The fact that the cell membrane phospholipids are the main source of light scatter in tissues and the lipid removal is a potential approach for increasing the tissue transparency. Several techniques of the lipid removing transparency have been developed for the 3D imaging of tissues, including using acrylamide protocols such as CLARITY (12), passive CLARITY (2), PACT, PARS (13), and also without applying acrylamide methods including FASTClear (14) and Fast Free-of-Acrylamide Clearing Tissue (FACT) (15). 

Some of these techniques use hydrogel embedding such as CLARITY and PACT. Not only are they costly, but they change the tissue volume even after using the refractive index matching solutions (RIMs). The complete tissue clearing needs several days to weeks to disrupt the fluorescent signal of chemically labeled proteins and it cannot finally prevent the quenching of fluorescent protein signals for a long time. These hydrogel-based techniques also need further toxic chemicals, labor work and the equipment. Therefore, a simple technique is appropriate for laboratories in developing countries. One of these newly-developed simple techniques is the FACT (15) requiring the lower labor work, and the use of toxic and environmentally hazardous chemicals in comparison to acrylamide-based protocols. Another limitation in the developing countries is the lack of advanced microscopes, i.e. confocal, 2-photon and light sheet microscopes. To date, all of the introduced protocols for the 3D imaging of tissues have used the advanced microscopes. Adopting FACT approach with a conventional epifluorescent microscope was another goal of this study.

Hopefully, this methodology may help in studying the brain vascular architecture for fundamental evaluation of pathological alterations in cerebral disorders including the vessels such as ischemia (16), Alzheimer’s disease (17), and cancer (18). Therefore, the aims of the present study were to evaluate the ability of the FACT protocol for clearing different whole tissue of mice and rats and 3D imaging of the brain cortex vasculature with FACT method in mice using a simple epifluorescent microscope in a non-developed imaging lab.

## Materials and Methods

### Animals

The present experimental study has been performed according to Shahid Sadoughi University of Medical Sciences Guidelines for Animal Handling and the Ethics Committee of Research and Clinical Center for Infertility (No: 91/8/2/2168). Adult female mice (n=3) and rats (n=3) were used and kept in Laboratory Animal Center of the Center of Infertility, Shahid Sadoughi University of Medical Sciences, Yazd, Iran.

### FACT protocol

**Fig.1:**
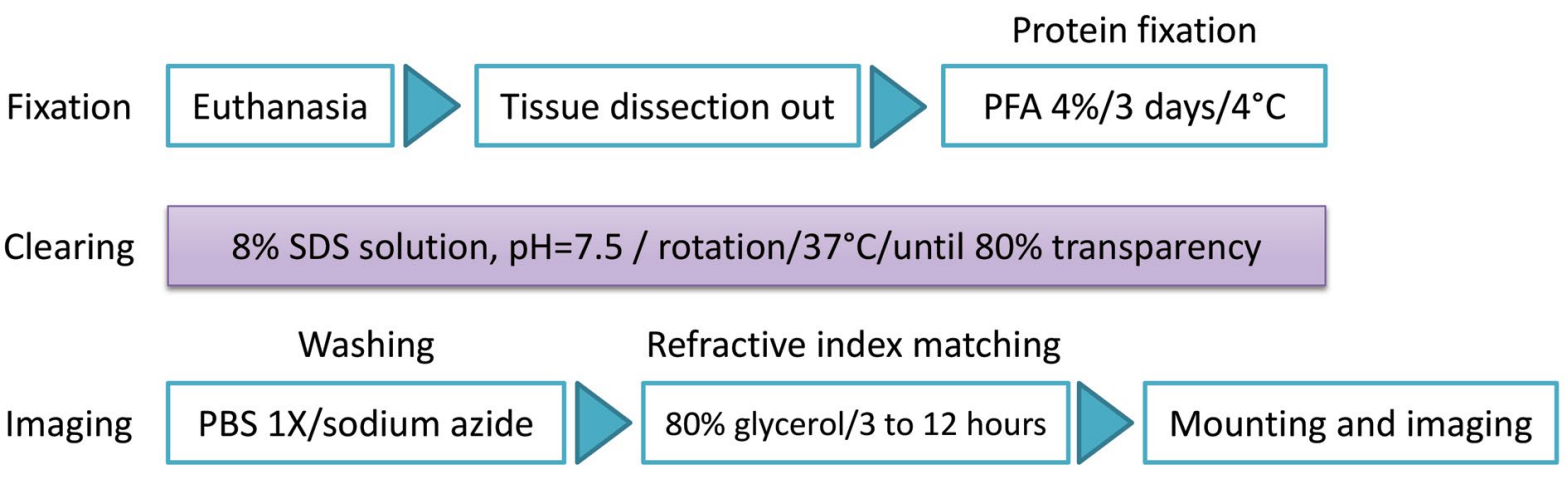
Protocol of clearing and imaging of brain cortex vasculature by Fast Free-of-Acrylamide Clearing Tissue (FACT) based on the presence of red blood cells and their auto-fluorescent signal. PFA; Paraformaldehyde, PBS; Phosphate buffer saline, and SDS; Sodium dodecyl sulfate.

The rats and mice were euthanized by ether inhalation and then cervical dislocation. The experiment protocol has been summarized in Figure 1. Then, tissues including the brain, spinal cord, heart, lung, adrenal gland, pancreas, liver, esophagus, duodenum, jejunum, ileum, skeletal muscle, bladder, ovary, and uterus were dissected out. They were separately transferred into 4% paraformaldehyde (PFA, Merck KCaA, Germany) diluted in phosphate- buffered saline (PBS, Gibco, UK) solution (0.01 M) as a fixative solution (pH=7.5, room temperature). Tissues were fixed in the fixative solution at 4°C for 3 days. Then, the brain was coronally 1 mm-sliced, coronally.

The whole and sliced tissues were cleared according to the FACT protocol (15). In details, the tissues were cleared with clearing solution containing 8% (wt/vol) sodium dodecyl sulfate (SDS) in 0.01 M PBS (pH=7.5) with 0.02% sodium azide at 37°C with mild rotational horizontal shaking (100 r/minutes) in a shaker incubator (Jaltajhiz, Iran). The clearing solution was refreshed daily for 3 days and then was replaced weekly until the visual confirmation of 80% tissue transparency by an observation through the tissue of clear black grid lines printed on a white paper. Transparency of the tissue during the clearing procedure was imaged using a DP71 camera (Olympus, Japan) on a stage of a loop microscope (SZX16, Olympus, Japan) for background illumination. The start and end date of clearing were recorded for all tissues. 

### Imaging of auto-fluorescent vessels in brain cortex

The brain slices were washed once in PBS with 0.02% sodium azide, and then were shaken gently 12 hours in the same solution at 37ºC in horizontally fixed falcon tubes. For complete transparency and refractive index (RI) matching, samples were placed in 80% glycerol in double distilled water for 3 to 12 hours in room temperature prior to imaging.

### 3D epifluorescent microscopy

For 3D imaging, brain slices were individually mounted between two glass slides which were surrounded by same thickness non-colorful putty that formed a horse-shoe-like chamber (1-mm thickness wall) to protect the tissues’ thickness from pressing between the slides and provides a chamber for the RI matching solution. This chamber between two slides was filled with fresh 80% glycerol. The auto- fluorescent vessels were imaged by an epifluorescence microscope (BX51 with a DP72 camera, Olympus, Japan), and CellSens imaging software (Version 1.4.1, Olympus, Japan). After the apparatus was fixed on the microscope stage, the specimen was imaged by an air/dry objective lens 10× (UPlanSApo, Olympus Co. Ltd.; numerical aperture : 0.4 and working distance: 3.1 mm) which was water immersed to increase working distance. The EPI illumination mode and red excitation (650 nanometers) and deep red emission (690 nanometers) were applied for imaging. For this purpose, selected area was imaged on a z-stack manner (each 10-µm step) for the depth of 150 µm from the tissue surface, automatically. 

### 3D image preparation

The TIFF image sequences were obtained from the microscope and transferred to Imaris software (version 7.4.2, ImarisX64, Bitplane AG) for the 3D reconstruction (19). In details, after importing TIFF files, in the "Display Adjustment" tabs, the color and name of the channels were changed. Then, in the drop-down list under the "Edit" button and “Image Properties” panel, the thickness of the tissue was corrected according to the z-stack imaging information of an epifluorescent microscope. To 3D crop, the final images and removing the excess parts, in the drop-down list under the "Edit" button and "Crop 3D" option were used. The size of the field was adjusted by dragging the borders. 

In the "Surpass" panel, vessels of brain cortex were 3D reconstructed using the filaments algorithm and based on the detected signals. In detail, a new Filament was created in the "Filaments" button. In the "Slice" panel, the thinnest and the largest diameter of the imaged vessels were defined. On the "Measure" panel, the distance automatically was shown after selecting two points at the maximum width of the thinnest and thickest vessels. After defining the largest and thinnest thickness, data were entered in the "Surpass" panel. Then, the thresholds of starting and seed points were adjusted. Tracing the length of vessels, using "Select" tab in the "Camera"panel, some of the automatically produced seed points were manually removed by pressing shift on the keyboard and left clicking on the point. The 3D image rotation was done by selecting "Navigate" and moving the pointer of the mouse device, to ensure that the correct seed points have been retained.

Then, the highest threshold for the local contrast was selected. At the last step, without the selection of "Detect Spines", the blood vessel reconstruction was finalized. The excess parts of the vessels which were not matched on the signals were removed in the "Edit" panel. The color of reconstructed cylinders was edited by clicking the "Color" tab.

### Comparing antibody stained and auto-fluorescent vessels 

Comparing the vessel imaging by non-antibody-based and antibody staining method, brain slices of non-perfused and perfused mice, respectively, were cleared using the FACT protocol. Then, both groups were labeled for CD31 (a marker of blood vessels epithelium) and Hoechst 33342 (marker of the cell nucleus).

In details, after clearing, the residual SDS was washed from the brains by slow shaking in PBS with 0.1% Triton X-100 (PBST) for 24 hours at 37°C. The samples were then incubated for 24 hours with antiCD31 primary antibody (1:10, mouse species, Abcam, USA) diluted in PBST with shaking at 37°C. The samples were subsequently washed in PBST buffer for 24 hours with shaking at 37°C. Then they were incubated with the FITC-IgG secondary antibody (Goat anti-mouse, 1:100, Abcam, US) diluted in PBST for 24 hours with shaking at 37°C in a tube was covered with an aluminum sheet. To label cell nuclei, Hoechst 33342 (1:100, Bis Benzimaide H 33342, Sigma, USA) was added to the secondary antibody mixture for the final 12 hours of incubation with shaking at 37°C. Before mounting and imaging, samples were washed in PBST for 24 hours with shaking at 37°C. Samples were submerged in glycerol for 24 hours at room temperature. The antibody signals and auto- fluorescent vessels were imaged by an epifluorescence microscope (BX51 with a DP72 camera, Olympus, Japan), and CellSens imaging software (Version 1.4.1, Olympus, Japan).

To evaluate the possibility of detection of vessels in other tissues by auto-fluorescence characteristics of the red blood cell (RBC), non-perfused spinal cord and uterus along with perfused skeletal muscle and duodenum of mice were stained with Hoechst 33342.

## Results

**Fig.2:**
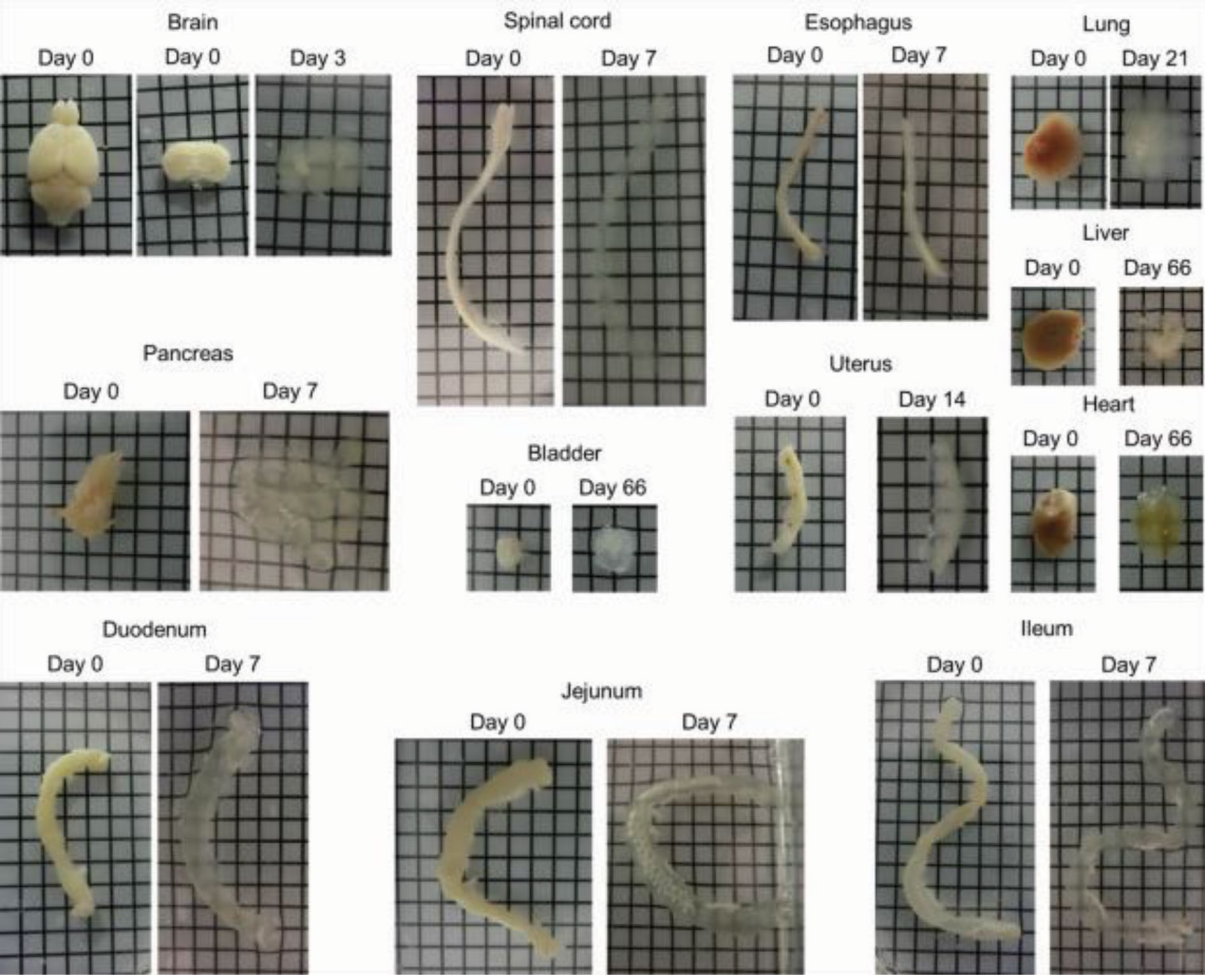
Clearing of mouse tissues using Fast Free-of-Acrylamide Clearing Tissue (FACT) including 1 mm brain slice, spinal cord, esophagus, lung, pancreas, bladder, uterus, heart, duodenum, jejunum, ileum (squares are 3×3 mm^2^). The clearing steps are before using refractive index solution (RIMs) and transparency is not more than 80%.

**Fig.3:**
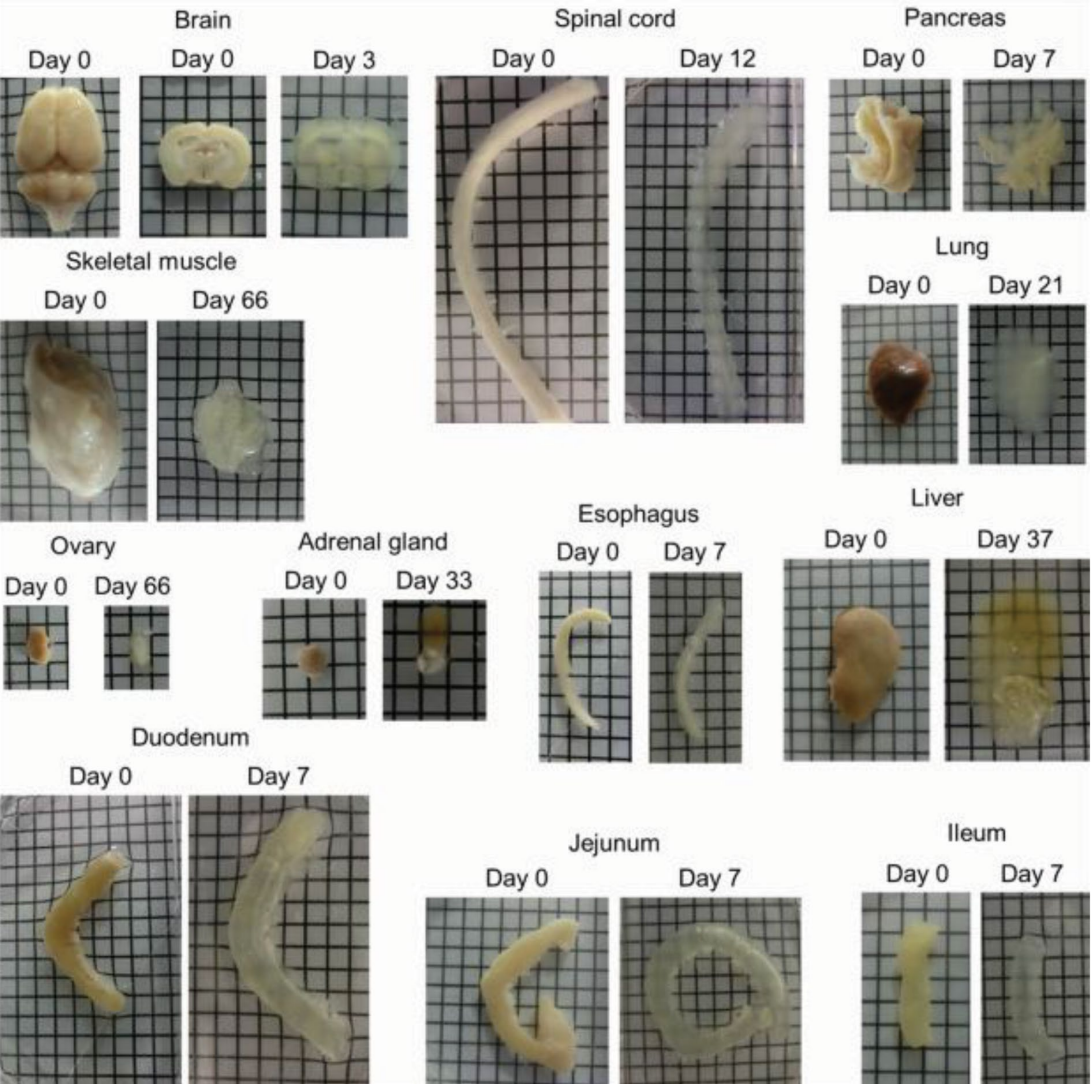
Clearing of rat tissues using Fast Free-of-Acrylamide Clearing Tissue (FACT) including 1 mm brain slice, spinal cord, pancreas, skeletal muscle, lung, ovary, adrenal gland, esophagus, liver, duodenum, jejunum, and ileum (squares are 3×3 mm^2^). The clearing steps are before using refractive index solution (RIMs) and transparency is not more than 80%.

Transparent brain slices were rapidly created with the passive clearing using the FACT protocol (Figes.2, 3). As shown in Figures 2 and 3, the FACT cleared the 1-mm thick brain slices in both mice and rats within 3 days (Table 1).

**Fig.4:**
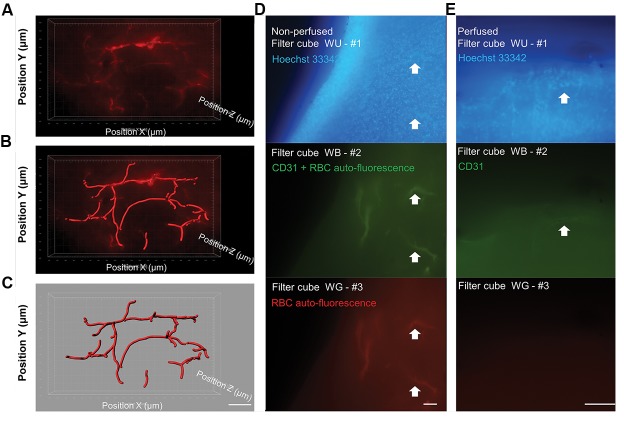
Fast Free-of-Acrylamide Clearing Tissue (FACT) technique for three-dimensional (3D) imaging of blood vessels in brain cortex using an epifluorescentmicroscope. A. A 3D image of the mouse blood vessels by the FACT clearing protocol. The fluorescent signal is from auto-fluorescent heme in red blood cells inthe non-perfused blood vessels (tissue dimension XYZ=2000×1200×150 µm^3^) (scale bar: 300 µm), B. Using Imaris software and “Filament” algorithm of “Surpass”, vessels’ structure was reconstructed (scale bar: 300 µm), C. 3D segmentation of cortex blood vessels in 150 µm depth of cortex (scale bar: 300 µm), and D, E. Comparison of imaging of brain cortex vessels by non-antibody-based and antibody staining method, in brain slices of non-perfused and perfused mice, D. In non- perfused brain similarity of the architecture of vessels were imaged with WB and WG filters demonstrates that RBC signals have been completely overlapped withCD31-labeled vasculatures (scale bar: 100 µm), E. In perfused mice, vessels are observable with WB filter. Arrows demonstrate vessels (scale bar: 100 µm).

To 3D reconstruct the blood vessels architecture in the brain cortex of mice, a microvasculature containing red blood cells was subsequently examined by an epifluorescent microscope (Fig .4A). In addition, using Imaris Filament algorithm, the blood vessels were segmented (Fig .4B). The 3D reconstructed blood vessels in brain cortex of mice were shown in Figure 4C. 

**Table 1:** Comparison of clearing time (day) of whole or sectioned tissues of mouse and rat with Fast Free-of-Acrylamide Clearing Tissue (FACT), passive CLARITY, PACT, and mPACT methods


Tissue	FACT		Passive CLARITY		PACT		mPACT	
	Mouse	Rat	Mouse	Rat	Mouse	Rat	Mouse	Rat

Brain slice (1-mm thickness)	3	3	4 (21)	6 (21, 25)	4-9 (13, 15)	ND	ND	ND
Spinal cord (whole size)	7	12	14-28 (24, 26)	ND	12 (30)	12 (30)	14 (30)	21 (30)
Heart (whole size)	66	ND	ND	ND	17 (30)	16 (30)	15 (30)	16 (30)
Lung (whole size)	21	21	30 (20)	ND	18 (30)	18 (30)	14 (30)	18 (30)
Adrenal gland (whole size)	ND	33	ND	ND	ND	ND	ND	ND
Pancreas (whole size)	7	7	ND	ND	15 (30)	15 (30)	17 (30)	15 (30)
Liver (one lobe)	6	37	30 (27)	ND	22 (30)	23 (30)	ND	23 (30)
Esophagus (whole size)	7	7	ND	ND	ND	ND	ND	ND
Intestine (whole size)	7	7	12-30 (20, 28)	ND	12-14 (28)	ND	ND	ND
Bladder (whole size)	66	ND	ND	ND	ND	ND	ND	ND
Ovary (whole size)	ND	66	35 (19, 29)	ND	ND	ND	ND	ND
Uterus (whole size)	14	ND	ND	ND	ND	ND	ND	ND


ND; No data.

The FACT protocol could make transparent the different types of tissues of mice (Fig .2) and rats (Fig .3). For the whole organs of adult female mice and rats, the optimal passive clearing conditions were determined for the FACT and compared with the previous studies in Table 1.

In order to show the possibility of vessels to be imaged by a non-antibody-based method based on auto-fluorescent characteristics of the RBC and to confirm detectability of the vessels in this protocol to be imaged after the FACT technique, the brain slices of non-perfused and perfused mice were stained in the same staining condition and the same tube containing CD31. Using blue (WB) and green (WG) filter boxes, the auto-fluorescent RBC signals were detected in non-perfused vessels of the brain cortex (Fig .4D). There were no vessels in the image of WB filter which were not visible in WG filter, too. This phenomenon showed that the auto-fluorescent RBC signals and CD31-labeled vascular endothelium were completely overlapped. In contrast, in perfused mice (Fig .4E), FITC-labeled CD31 markers on vascular endothelium were only observable in WB filter. In the both non- perfused and perfused mice, vascular endothelium nuclei line-shaped structures were visible by Hoechst 33342 staining.

**Fig.5:**
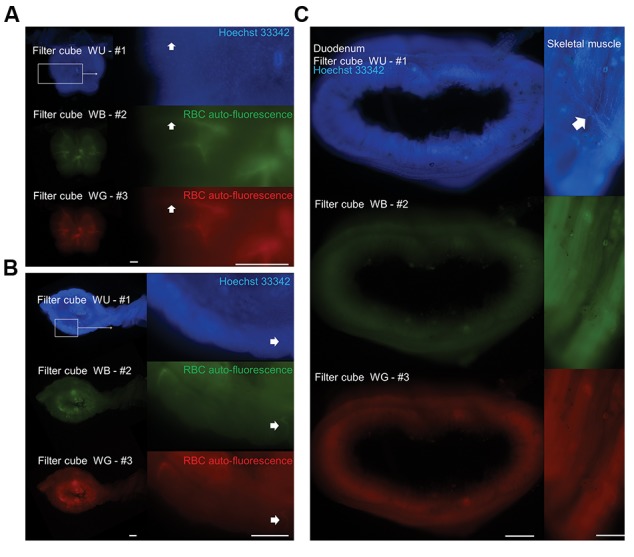
Vascular imaging after Fast Free-of-Acrylamide Clearing Tissue (FACT) technique. A. Imaging of spinal cord vessels by a non-antibody-based method in the non-perfused mice (scale bar: 200 µm). Arrows demonstrate auto-fluorescent vessels, B. Imaging of vessels in the uterus and attached broad ligament by a non-antibody-based method in the non-perfused mice (scale bar: 200 µm). Arrows demonstrate auto-fluorescent vessels, and C. Imaging ofduodenum and skeletal muscle in the perfused mice after clearing by Fast Free-of-Acrylamide Clearing Tissue (FACT) technique and labeling with Hoechst 33342 (scale bar: 400 µm). Arrow alludes to vascular branches.

Using a similar method of non-antibody-based detection of vessels, we evaluated the imaging of the FACT-cleared spinal cord (Fig .5A) and uterus (Fig .5B) in non-perfused mice. Furthermore, imaging of duodenum and skeletal muscle in perfused mice after clearing by the FACT technique did not show any vascular structures (Fig .5C). 

## Discussion

For the first time, we demonstrated the clearing of the murine tissues with the FACT protocol (15) for the effective clearing of tissues and 3D imaging of brain cortex vasculatures. The FACT method, has been modified including alterations of imaging to adapt this method for non-equipped laboratories. Although the most important part of the whole tissue clearing is optical sectioning for 3D imaging and this can be optimally achieved by confocal microscopy, availability of this expensive microscope is a big challenge for adapting the whole tissue imaging for a conventional laboratory of limited resources. Therefore, in the present study of the FACT method we used an epifluorescent microscope with a motorized stage for imaging auto-fluorescent vessels in the z-plane. Bearing in mind that this approach has some limitations including a lower depth of imaging of fluorescent light in an epifluorescent microscope in comparison to a laser in a confocal microscope. This issue can be solved by cutting 1- to 2-mm piece of cleared tissue for imaging. Moreover, a lower power of the epifluorescent for collecting the enhanced signals in comparison to the laser-enhanced fluorophores in confocal caused limitation in imaging of tissue for the maximum depth of 200 to 300 µm.

The FACT cleared 1-mm of thick brain slices in both mice and rats in 3 days, while in mice and rats a passive CLARITY needs 4 and 6 days, respectively and PACT requires 9 days in mice . Regarding the clearing time, the whole brain slice (1 mm thickness) clearing in murine species with the FACT protocol as a passive method required 3 days for completion which is comparable to CUBIC (more than 1 week) (9) and ScaleA2 (5 days) (4). In addition, based on Xu et al. (15) and comparing of our data with previous studies, we can speculate that removing hydrogel decreased the clearing time in comparison to hydrogel-based methods such as CLARITY (2), PACT, PARS (13), and SWITCH (10). The CLARITY technique has been used for clearing the different murine tissues including the brain (2, 20-25), spinal cord (21, 24, 26), heart (22), lung (20, 22), liver (27), intestine (20, 28), skeletal muscle (22), and ovary (19, 29). In addition, several studies cleared different murine tissues using PACT protocol including the brain (13, 22, 30, 31), heart, lung (13, 22, 30), pancreas, liver (30), intestine (13, 28), skeletal muscle (22), adrenal gland (20), and ovary (30). In addition to above-mentioned tissues which were cleared by acrylamide-based protocols (32), using FACT protocol, the other murine tissues including bladder, uterus, and esophagus have been cleared for the first time in this study. 

Brain vasculature imaging at microscopic scales and imaging deep into brain remained an open quest in neuroscience. Although, the conventional optical microscopy is still limited to surface imaging. Revolutionary approaches such as ultrasonography (33), intravital microscopy (34), and the whole tissue imaging by an optical microscope (35) have opened new windows in this aspect. The whole tissue clearing such as our findings, can show the 3D architecture of the blood vessels and also can be used for seeking vasculature relationships in neurons (23). 

Epifluorescent, confocal and light sheet microscopy provide more information for the vasculature research in the brain because of the presence of various vascular endothelial markers such as CD31, CD34, factor VIII, von Willebrand factor, and Fli-1 that can be used in these imaging methods especially, in whole tissue imaging (36). In addition to mentioned markers which can be detected by chemical or genetic labeling methods for the detection of brain vasculature (36, 37), other fluorescent materials have been also used for imaging of the brain vasculature (38). In the present study, we used the auto- fluorescent character of heme in red blood cells (39) in a non-perfused brain for the visualization of the blood vessels in the FACT-cleared mouse brain, spinal cord, and uterus.

## Conclusion

The FACT method is a simple technique which might be appropriate for laboratories in developing countries lacking advanced microscopes such as confocal microscopes. Successful labeling the vessels in murine species after clearing by the FACT approach resulted in 3D imaging of brain cortex vessels for the first time. Imaging the vasculature was performed without any staining; rather accomplished by a simple auto-fluorescence imaging of the RBC. 
